# A Toxin–Antitoxin System Promotes the Maintenance of an Integrative Conjugative Element

**DOI:** 10.1371/journal.pgen.1000439

**Published:** 2009-03-27

**Authors:** Rachel A. F. Wozniak, Matthew K. Waldor

**Affiliations:** 1 Channing Laboratory, Brigham and Women's Hospital, Harvard Medical School, Boston, Massachusetts, United States of America; 2 Howard Hughes Medical Institute, Chevy Chase, Maryland, United States of America; 3 Program in Genetics, Tufts University School of Medicine, Boston, Massachusetts, United States of America; Universidad de Sevilla, Spain

## Abstract

SXT is an integrative and conjugative element (ICE) that confers resistance to multiple antibiotics upon many clinical isolates of *Vibrio cholerae*. In most cells, this ∼100 Kb element is integrated into the host genome in a site-specific fashion; however, SXT can excise to form an extrachromosomal circle that is thought to be the substrate for conjugative transfer. Daughter cells lacking SXT can theoretically arise if cell division occurs prior to the element's reintegration. Even though ∼2% of SXT-bearing cells contain the excised form of the ICE, cells that have lost the element have not been detected. Here, using a positive selection-based system, SXT loss was detected rarely at a frequency of ∼1×10^−7^. As expected, excision appears necessary for loss, and factors influencing the frequency of excision altered the frequency of SXT loss. We screened the entire 100 kb SXT genome and identified two genes within SXT, now designated *mosA* and *mosT* (for maintenance of SXT Antitoxin and Toxin), that promote SXT stability. These two genes, which lack similarity to any previously characterized genes, encode a novel toxin-antitoxin pair; expression of *mosT* greatly impaired cell growth and *mosA* expression ameliorated MosT toxicity. Factors that promote SXT excision upregulate *mosAT* expression. Thus, when the element is extrachromosomal and vulnerable to loss, SXT activates a TA module to minimize the formation of SXT-free cells.

## Author Summary

Integrative and conjugative elements (ICEs) are a diverse group of mobile genetic elements found in many bacteria. These elements integrate into the host chromosome as well as excise and transfer to other bacteria. SXT, an ICE that encodes resistances to multiple antibiotics, is currently present in most clinical isolates of *V. cholerae*, the cause of cholera. Cells in which SXT is excised can potentially lose the ICE if cell division occurs prior to its re-integration, but explorations of mechanisms that promote ICE maintenance have received almost no attention. Using a positive selection-based strategy to detect SXT loss, we found that loss of this ICE was very rare (1 in 10^7^ cells). Two genes, *mosAT*, that lack similarity to genes of known function were found to promote SXT maintenance. We show that MosT blocks cell growth in the absence of SXT and its activity can be neutralized by MosA. Thus, these genes encode a functional toxin–antitoxin (TA) system. When SXT is extrachromosomal and vulnerable to loss, *mosAT* expression increases, minimizing the formation of SXT-free cells. The activity of *mosAT* may contribute to the maintenance of antibiotic resistance in bacterial populations.

## Introduction

Integrating and conjugative elements (ICEs) are a class of self-transmissible mobile genetic elements that contribute to horizontal gene exchange among bacteria [1]. With our increasing knowledge of bacterial genomes, ICEs and putative ICEs have been identified in the chromosomes of many gram-positive and gram-negative bacteria [1]–[7]. ICEs have some features in common with plasmids and bacteriophages, other classes of mobile genetic elements that have been the subjects of more study. Like many temperate bacteriophages, ICEs integrate into and replicate with the host chromosome. However, like conjugative plasmids, ICEs encode DNA processing and type IV secretion machinery that enables their conjugative transfer from donor to recipient cells. ICEs excise from the host chromosome to form an extra-chromosomal circular molecule that is thought to be the substrate for conjugative transfer. Although the excised circular ICE form is presumed to undergo rolling-circle replication concomitant with its transfer from donor to recipient cell, ICEs, unlike plasmids, are not thought to be ordinarily capable of autonomous replication. In addition to diverse integration, excision and conjugation systems, ICEs encode a variety of additional properties including resistance to antibiotics [8]–[10], nitrogen fixation [11], and degradation of aromatic compounds [12].

SXT is a ∼100 Kb ICE originally discovered in a clinical isolate of the cholera pathogen, *Vibrio cholerae* [13]. SXT and closely related ICEs encode resistances to antibiotics such as sulfamethoxazole and trimethoprim that have previously been useful for the treatment of cholera. In the past 15 years, SXT-related ICEs have become widespread in clinical *V. cholerae* isolates in Asia and Africa [14]. SXT-related ICEs have also been isolated from other bacterial species; for example, R391, an Inc J element derived from *Providencia rettgeri* [15], was found to be genetically and functionally highly similar to SXT [8],[16],[17]. Currently, there are more than 25 known members of the SXT/R391 family of ICEs [14]. These ICEs are all thought to consist of a conserved set of core genes that mediate the elements' integration, excision, conjugation and regulation [18]. Besides the conserved core genes, these ICEs also contain element-specific genes, conferring attributes such as resistance to heavy metals or antibiotics; however, the function of most non-core SXT genes remains unknown.

All SXT/R391 family ICEs encode nearly identical tyrosine recombinases (Int) that mediate the integration of the respective ICEs within the *prfC* locus in the host chromosome. Int mediates the site-specific recombination between a site (*attP*) found in the circular form of the ICE and a site (*attB*) found near the 5′ end of *prfC*. In addition, these ICEs all encode similar Xis proteins, which act along with Int to promote the elements' excision from the chromosome. Excision occurs by Int-mediated recombination between short sequences found at the right and left ends of the integrated element, yielding the extrachromosomal circular form of the ICE [19]. Xis functions as a recombination directionality factor (RDF), promoting SXT excision and inhibiting its integration [20].

SXT transfer functions are not constitutively expressed. An SXT-encoded repressor, SetR, ordinarily limits SXT transfer. SetR, a homologue of the lambda phage repressor, cI, represses the expression of SetC and SetD, transcription factors that activate expression of *int* and the *tra* operons that encode the proteins required for SXT's conjugative transfer [18],[21],[22]. DNA-damaging agents, including certain antibiotics, increase the frequency of SXT transfer by alleviating SetR repression of *setDC* expression, probably by promoting RecA-dependent SetR autoproteolysis [22].

In host cells that contain an ICE, there is an equilibrium between the integrated and excised circular ICE forms. For example, in the case of *E. coli* containing SXT, we found that ∼2% of cells in the population harbored excised SXT, detected as an amplifiable chromosomal *attB* site by quantitative PCR [20]. SXT excision from the chromosome poses a potential threat to the element's stability: if the host divides while SXT is extrachromosomal, the element may not be passed to a daughter cell. Our anecdotal observations suggest that cells rarely if ever lose SXT raising the possibility that this ICE may encode mechanisms to promote its maintenance. To date, there have been no systematic studies of mechanisms that promote ICE maintenance. Here, we developed a positively selectable reporter of SXT loss to begin to explore the environmental and genetic factors that influence this ICE's stability in *E. coli*. Using this reporter, SXT loss was detected in 1×10^−7^ cells. Excision appears necessary for loss, and factors influencing the frequency of excision altered the frequency of SXT loss. We screened the entire 100 kb SXT genome and identified two genes, which lack similarity to genes of known function that promote SXT maintenance. These genes, designated *mosA* and *mosT*, encode a toxin-antitoxin (TA) system. We found that factors that promote SXT excision also augment *mosAT* expression. Thus, when the element is extrachromosomal and vulnerable to loss, SXT activates a TA module to minimize formation of SXT-free cells.

## Results

### A Positively Selectable Reporter of SXT Loss

**Figure 1:**
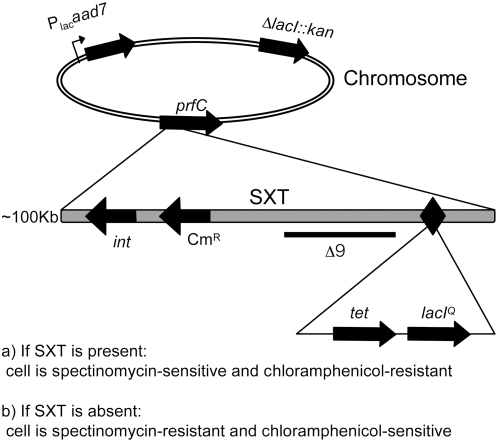
Schematic of a positively selectable reporter of SXT loss. SXT containing *lacI^Q^* was introduced into a *lacI E. coli* host containing Plac*-aad7* (a spectinomycin-resistance gene). If SXT (and *lacI^Q^*) is lost, the cells become Spec^R^. The black diamond represents the site of insertion for a fragment containing *lacI^Q^* (pFRTIq), thick black arrows represent ORFs, and the thin black line indicates the approximate location of the Δ9 deletion. Abbreviations: *tet* = tetracycline resistance gene, *kan* = kanamycin resistance gene, *int* = integrase, *prfC* = site of SXT insertion. Figure not to scale.

During serial passage of SXT-bearing strains without selection for SXT-encoded markers, we never detected cells that have lost this ICE, suggesting that SXT is stably maintained in the host population. Given the apparently low frequency of SXT loss, an *E. coli* reporter strain was designed to allow positive selection of rare cells that have lost SXT (Figure 1). To construct this strain, the Lac repressor, *lacI^Q^*, was introduced into an innocuous location in SXT (between *traG* and *s079*) while the native *lacI* was deleted from the chromosome. Additionally, a spectinomycin (Spec) resistance cassette under the control of the *lac* promoter was introduced into the *gal* locus in the chromosome (Figure 1). Therefore, in the presence of SXT (and therefore *lacI^Q^*), cells are sensitive to spectinomycin. If SXT (and *lacI^Q^*) are lost, cells become resistant to spectinomycin. As a result, even very rare cells lacking SXT can be detected as Spec-resistant colony forming units (cfu). Once Spec-resistant cfu are identified, they can subsequently be screened for the presence of SXT-encoded antibiotic resistances, such as chloramphenicol, to confirm SXT loss.

### Detectable SXT Loss Is Rare

**Figure 2:**
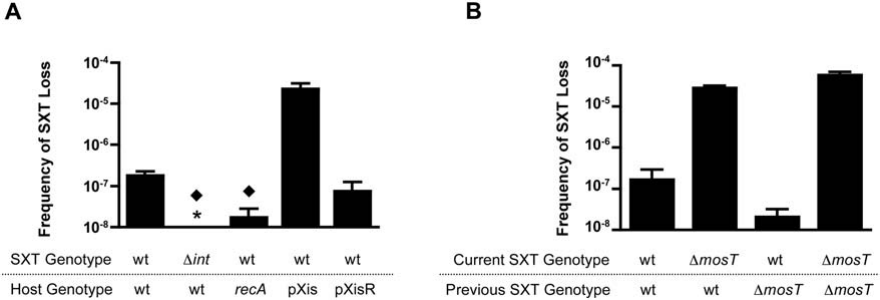
Influence of genetic factors on the frequency of SXT loss. The frequency of SXT loss was calculated as the ratio of Spec^R^Cm^S^ cfu / total cfu after 15 hour of growth. A) Factors that influence the frequency of SXT excision alter the frequency of SXT loss. The black diamonds indicate that the result is statistically different (p<0.05) than the result shown in the first column for the wild type reporter. The * signifies that the result was below the limit of detection which was ∼1×10^−8^. pXis and pXis-R harbor arabinose-inducible *xis* or its reverse complement respectively. B) SXT loss is not accompanied by heritable changes that influence maintenance of SXT. The ‘current SXT genotype’ refers to the ICE introduced into the host that lost the ICE designated as ‘previous SXT genotype’.

Loss of SXT from the *E. coli* reporter strain described above was detected at a very low frequency. After 15 hours of growth (∼20 generations) without selection for SXT-encoded markers, only ∼1×10^−7^ cfu were Spec-resistant and Cm-sensitive, indicating that they had lost SXT (Figure 2A). Similar extremely low levels of SXT loss were observed in exponential and in stationary phase cultures (data not shown), suggesting that culture growth phase does not influence SXT loss. This apparently very low frequency of SXT loss explains why we did not previously detect loss in replica-plating based screens for cells that lost resistance to SXT-encoded antibiotic markers. To confirm that SXT loss was not accompanied by heritable changes in the host chromosome, SXT was re-introduced into the reporter strain that had previously lost this ICE. SXT was lost from this re-constituted SXT loss reporter strain at approximately the same frequency as the original reporter (compare the first bars in Figure 2A and 2B), suggesting that loss of the element was not associated with mutations in the host chromosome.

### Excision and Loss

To begin to explore the relationship between SXT loss and its excision from the chromosome, we assessed the frequency of SXT excision in our reporter strain using quantitative PCR. In this assay, the amount of *attB*, which is only generated if SXT excises from the chromosome, is compared to the amount of a stable chromosomal locus (the 3′end of *prfC*), as described in Burrus and Waldor [20]. We found that SXT excises in 3–4% of cells in the population, a frequency that is nearly 100,000 times the frequency of detectable SXT loss (Table 1). SXT excision may routinely be followed by its re-integration. Furthermore, there may be mechanisms for either preventing DNA replication/cell division when SXT is excised and/or selecting against cells that have lost SXT. Overall, it appears that excision events are only rarely associated with formation of cells that can grow despite losing SXT; however, these numbers may underestimate the true extent of SXT loss if there is selection against such cells.

**Table 1:** Comparison of SXT excision and loss frequencies.

SXT^1^	Excision Frequency^2^	Loss Frequency^3^	Loss Events/Excision Events
Wild type	3.7×10^−2^	3.2×10^−7^	8.5×10^−6^
Δ*mosT*	3.8×10^−2^	3.3×10^−5^	8.7×10^−4^

1 Isogenic *E. coli* hosts (RF146 or RF335) harboring either wild type SXT or *most* SXT were used in these assays.

2 Calculated using QPCR by measuring the ratio of *attB*: 3′*prfC* (see [20]).

3 Calculated as the fraction of Spec^R^ cfu/total cfu.

Since the SXT integrase is required for the element's excision from the chromosome [19],[20], we deleted the *int* gene from the SXT loss reporter strain to test if SXT loss requires its excision. SXT loss was never observed in the Δ*int* SXT strain (Figure 2A) suggesting, as expected, that SXT excision is required for its loss. However, it is important to note that the limit of detection for this assay is ∼1×10^−8^, and thus can only conclude that SXT loss is reduced at least 10-fold in the Δ*int* background.

There appears to be some additional correlation between the frequency of SXT excision and its loss. In a *recA* background, where SXT excision is reduced (data not shown), there was a statistically significant reduction in SXT loss (Figure 2A). Conversely, increased SXT excision promoted its loss. Overexpression of Xis, the SXT recombination directionality factor (RDF), which increases the frequency of excised SXT (unpublished observations), resulted in an ∼500-fold increase in SXT loss (Figure 2A, pXis bar). A control plasmid containing *xis* in the reverse orientation (pXisR) did not alter SXT loss (Figure 2A). As an RDF, Xis could promote SXT loss by both promoting its excision and inhibiting its reintegration into the host chromosome.

### Conjugation Does Not Lead to SXT Loss

Using the SXT loss reporter strain as a donor in conjugation experiments, we were able to address a longstanding question regarding SXT: do donors lose this ICE during conjugation? Following a 2 hour mating between the SXT loss reporter with an *E. coli* recipient, cells were plated on selective media to score for the number of donors, recipients, and exconjugants, as well as loss of SXT from the donor. As a control, the donor was grown by itself in identical conditions. As shown in Table 2, the frequency of exconjugant formation in this experiment was more than four orders of magnitude greater than the frequency of detectable SXT loss from the donor strain. Furthermore, the frequencies of SXT loss from the donor that was mixed with the recipient and the donor grown by itself under identical conditions were fairly similar. Although not statistically significant, there appears to be less loss of SXT when the donor was mixed with the recipient (Table 2). Together these observations suggest that donors do not lose SXT during conjugation and that a copy of SXT re-integrates into the donor chromosome following transfer of the ICE to the recipient.

**Table 2:** Conjugation does not increase SXT loss.

Donor^1^	Recipient	Loss of Donor^2^	Exconjugant Formation^3^
SXTwt	None	5.4×10^−7^	N/A
SXTwt	MG1655	3.3×10^−8^	3.1×10^−4^

1 SXTwt is strain RF146.

2 Loss was calculated as indicated in materials and methods.

3 Exconjugant formation was calculated as the number of exconjugant cfu/number of donor cfu. The data is from a representative experiment.

### Identification of SXT Genes That Promote Its Maintenance

Given the very low frequency of SXT loss, we hypothesized that SXT contains genes that promote its maintenance in the host. We took advantage of a set of previously constructed SXT deletion mutants [18] and constructed new deletion mutants to screen for regions of this ICE's genome that contribute to its stability. The features of the SXT loss reporter as outlined in Figure 1 were introduced into 11 SXT deletion mutants spanning the entire SXT genome, yielding 11 new SXT loss reporter strains (Table 3). Each of the new reporters was tested for SXT loss and the findings from these experiments are summarized in Table 3.

**Table 3:** Summarized deletions tested for SXT stability.

Deletion	Number of ORFs	Length of Deletion (bp)	Deletion Contents	Increased Frequency of SXT Loss^1^
Δint	1	1239	Integrase (*int*)	N
Δ1	20	19993	transposon-like genes *tnp*, *tnpA*, *tnpB*, *tnpB′*, *tnpA′*; antibiotic resistances *floR*, *dfr18*, *strA*, *strB*, *sulIII*; UV repair homologues *rum′B*, *rumB′*, *rumA*; 5 hypothetical genes	N
Δ2	17	20131	17 hypothetical genes	N
Δ3	1	2148	conjugative transfer gene *traI*	N
Δ4	2	3009	conjugative transfer gene *traD*; 1 hypothetical gene	N
Δ7	2	320	hypothetical toxin-antitoxin *s044-45*	N
Δ8	6	4109	conjugative transfer genes *traL*, *E*, *B*, *V*, *A*; 1 hypothetical gene	N
Δ9	8	11624	conjugative transfer genes *traC*, *F*, *W*, *U*, *N*; DsbC-like gene (*s054*); *mosA*, *mosT*	Y
Δ10	14	15112	*bet*, *exo*, and *ssb* homologues; 11 hypothetical genes	N
ΔD	5	11378	transfer genes *traF*, *H*, *G*; 2 hypothetical genes	N
ΔE	8	3669	entry exclusion gene (*eex*); master regulators *setC*, *setD*; master repressor, *setR*; 5 hypothetical genes	N

1 N, no elevation in the frequency of loss of the mutant SXT vs wt SXT; Y indicates at least an ∼5-fold increase in loss relative to wild type SXT.

Nearly all of the mutants tested exhibited rare loss of SXT, very similar to the loss frequency for wild type SXT. Several mutants (Δ3, Δ4, Δ8 and ΔE) contained deletions in genes required for conjugative transfer [18]; however, these mutants did not exhibit decreased SXT loss (Table 3) as would be expected if conjugation promoted SXT loss. Two deleted regions, Δ2 and Δ10, primarily consisted of predicted open reading frames of unknown or conserved hypothetical function, but these regions did not contain genes important for SXT maintenance (Table 3). Finally, two deletions that might have been suspected to influence SXT stability, Δ1 and Δ7, did not. The Δ1 deletion includes several transposases but deletion of this region did not promote SXT instability. The two genes deleted from the Δ7 mutant, *s044-45*, were previously proposed to function as a toxin-antitoxin module in SXT based on homology to a TA pair encoded in the *Paracoccus aminophilus* plasmid pAMI2 [23]. Our observation that the Δ7 mutant did not exhibit increased SXT loss argues against the idea that these genes encode a functional TA module in SXT.

**Figure 3:**
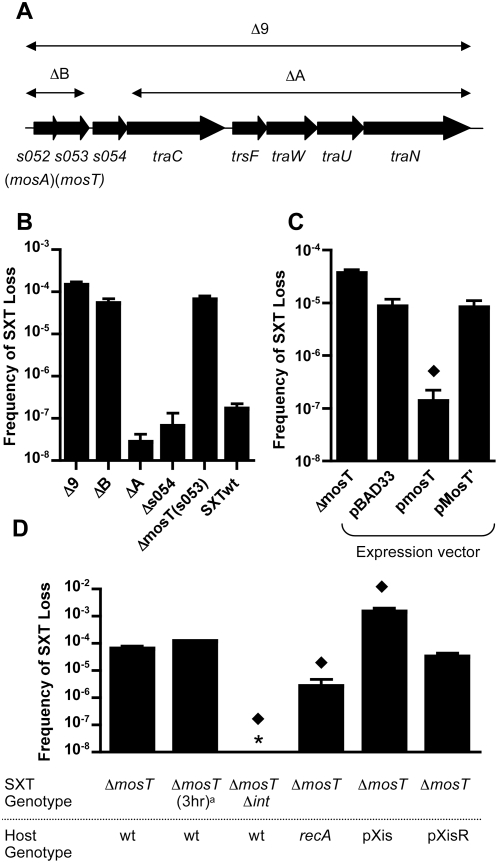
Genetic analysis of loss of *mosT* SXT. A) Schematic of the region deleted from Δ9 SXT. Black arrows represent ORFs, thin black arrows represent the deletions studied below. B) Frequency of loss of the indicated mutant SXT. C) Influence of *mosT* expression *in trans* on the stability of *mosT* SXT. All cultures were grown in the presence of 0.02% arabinose for 15 hr. Black diamond represents a statistically significant (p<.05) result compared to loss in the presence of pBAD33. D) Loss of *mosT* SXT is influenced by factors affecting SXT excision. All cultures were grown for 15 hr except as noted. Black diamonds represent a statistically significant (p<0.05) result compared to the *mosT* SXT grown for 15 hr. The * signifies that the result was below the limit of detection which was ∼1×10^−8^. ^a^Loss was calculated following 3 hours of growth in an early log phase culture.

However, deletion of the 8 genes in the Δ9 mutant, which spans the region from *s052* to *traN*, resulted in growth of markedly more colonies that lacked SXT (∼1×10^−4^), than observed to lack wt SXT (∼1×10^−7^) (Table 3 and Figure 3B). Five of the eight genes (see Figure 3A) in this deletion (*traC*, *trsF*, *traW*, *traU*, *traN*) are important for conjugative transfer of SXT. *s054* encodes a homologue of a disulfide bond isomerase-related protein and *s052* and *s053* are currently classified as hypothetical genes with no known function, characterized homologues, or recognizable motifs. We constructed smaller deletions within the *s052-traN* region to pinpoint those gene(s) that account for the increased loss observed for the Δ9 mutant. As expected, ΔA, which only removed genes implicated in conjugative transfer, did not alter SXT stability (Figure 3B); deletion of *s054* did not influence SXT stability either. However ΔB, which contains *s052* and *s053*, resulted in a similar frequency of loss as the Δ9 mutant. Finally, deletion of *s053* alone was sufficient to recapitulate the Δ9 phenotype (Figure 3B). It is important to note that while *s052* and *s053* could be deleted together, *s052* could not be deleted in the presence of *s053*. From this point on, for reasons that will become clear below, *s052* and *s053* are referred to as *mosA* and *mosT*, respectively, for maintenance of SXT (Antitoxin, Toxin).

### MosT Acts *In Trans* and Appears to Function following SXT Excision

We confirmed that the elevated frequency of loss of *mosT* SXT was due to the absence of *mosT* and not to a secondary mutation on the chromosome or in SXT in several ways. First, a *mosT* SXT as well as a wild-type SXT were reintroduced into a strain that had previously lost *mosT* SXT. In these two new strains, the frequency of loss of the respective ICEs was very similar to that observed in their original hosts (compare Figure 2B, 3^rd^ and 4^th^ bars vs. Figure 2A 1^st^ bar and 3B 5^th^ bar respectively), demonstrating that the high frequency of *mosT* SXT loss is not due to changes in the chromosome of its original host. Furthermore, the frequency of loss of *mosT* SXT from an *E. coli* strain that had previously lost wild type SXT was approximately the same as observed in the original host (Figure 2B, 2^nd^ bar vs 3B, 5^th^ bar), lending support to the idea that the elevated frequency of loss of *mosT* SXT is linked to this ICE. Finally, introducing the *mosT* deletion into a wild type *E. coli* SXT loss reporter strain, using either P1 transduction or λ Red-based technology [24] resulted in *mosT* SXT strains that had frequencies of *mosT* SXT loss that were the same as observed in our original mutant (data not shown). Together, these observations link the elevated frequency of *mosT* SXT loss to the absence of *mosT*.

To test whether *mosT* could act in trans to complement the elevated loss phenotype of *mosT* SXT, we constructed an arabinose-inducible version of *mosT* in the low copy vector pBAD33. Loss of *mosT* SXT was measured under inducing conditions in the presence of either the *mosT*-expression vector, pMosT, pBAD33 (empty vector), or pMosT', a pMosT derivative that was engineered to contain a stop codon in the 11^th^ codon of *mosT*. As shown in Figure 3C, expression of *mosT* from pMosT restored the stability of *mosT* SXT to levels observed in wild type SXT. In contrast, pMosT' did not alter the ICE's stability (compare 1^st^ and 3^rd^ bars Figure 3C). These observations suggest that *mosT* encodes a protein that promotes SXT stability.

To begin to explore how *mosT* influences SXT stability, we looked for conditions that alter the loss of *mosT* SXT. As with wild type SXT, the frequency of *mosT* SXT loss was not altered by growth phase (Figure 3D). MosT appears to act after SXT excises from the chromosome, since the frequency of *mosT* SXT excision was almost identical to that of wild type SXT (Table 1). Consistent with this idea, we found that *mosT* SXT loss was responsive to factors that influence SXT excision (Figure 3D) as observed above for wild type SXT. For example, loss of *mosT* SXT was abolished when *int* was deleted from *mosT* SXT and was reduced when a *recA* mutation was introduced into the host chromosome (Figure 3D and data not shown). Conversely, increasing *mosT* SXT excision by overexpression of Xis resulted in a significant increase in *mosT* SXT loss, whereas the control vector, pXis-R, did not alter *mosT* SXT loss (Figure 3D). Overall, the effects of *mosT* and *xis* appear to be independent, providing support for the idea that *mosT* likely acts after excision.

### MosT Inhibits Growth of *E. coli* and *V. cholerae*

**Figure 4:**
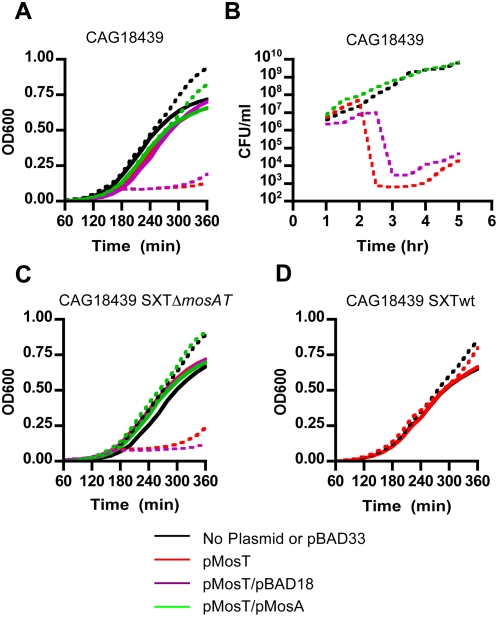
MosT inhibits *E. coli* growth and its toxicity can be neutralized by MosA. Growth kinetics of *E. coli* strains CAG18439 (A and B), CAG18439 containing *mosAT* SXT (C) and CAG18439 containing wild type SXT (D). These strains, which harbored arabinose-inducible *mosT* and *mosA*, (pMosT or pMosA respectively) or control vectors, (pBAD33 and pBAD18), were grown in either 0.2% glucose (solid lines) or 0.02% arabinose (dashed lines).

Our observation that *s052* (*mosA*) could not be deleted in the presence of *s053* (*mosT*) but that *mosT* could be deleted regardless of the presence of *mosA* suggested the possibility that *mosT* and *mosA* might comprise a toxin-antitoxin (TA) pair. TA pairs were originally described in plasmids but are now known to be present in many chromosomes as well [25],[26]. The toxin components of plasmid-borne TA modules are more stable than their cognate antitoxins; thus, cells that fail to inherit plasmids and can no longer synthesize antitoxin are thought to die or have impaired growth due to residual toxin activity [25],[27]. Several functions of chromosomal TA modules, including stress responses [28],[29] and stabilization of large chromosomal regions [30],[31], have been proposed. Consistent with the idea that *mosT* encodes a toxin, we found that *mosT* could only be cloned downstream of the arabinose-inducible promoter *P*_BAD_ in the presence of glucose, which represses *P*_BAD_ [32]. Furthermore, in the presence of arabinose, growth of *E. coli* harboring this plasmid-borne inducible *mosT* (pMosT) was severely impaired. There was minimal increase in culture OD600 following MosT induction (Figure 4A) and induction caused cfu to drop by several orders of magnitude (Figure 4B). Together, these data suggest that *mosT* over-expression is toxic to *E. coli* and *V. cholerae* (data not shown).

### MosA Can Neutralize MosT

**Figure 5:**
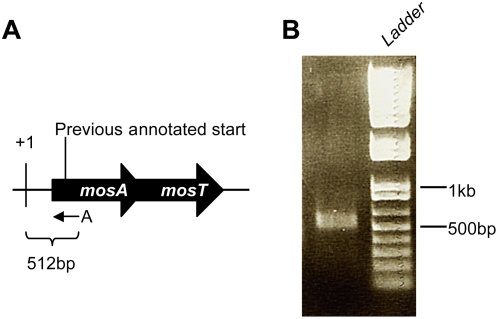
5′RACE analysis of *mosA* transcription start site. A) Schematic of *mosA* transcription start site based on the 5′ RACE results shown in B). The +1 refers to the start of transcription as defined by the DNA sequence of the 5′RACE product obtained using primer A (small black arrow). The length of the PCR product is indicated. Black arrows represent ORFs as predicted using bioinformatics; the previously annotated *s052* start codon is indicated. B) Shows a 1% agarose gel of the product of 5′RACE reaction using primer A.

Over-expression of MosT caused similar growth inhibition in *E. coli* lacking SXT (Figure 4A) and *E. coli* harboring *mosAT* SXT (Figure 4C), but had no effect on growth of *E. coli* harboring wild type SXT (Figure 4D), suggesting that *mosA* can neutralize the toxic effects of over-expressed MosT. To assess this possibility, we generated an arabinose-inducible *mosA* construct (in pMosA1). However, we were unable to demonstrate *mosA* neutralizing activity using this initial construct. Subsequent studies revealed that the putative *mosA* open reading frame was mis-annotated. 5′ RACE experiments to identify the +1 nucleotide of the *mosA* transcript (Figure 5B) suggested that the true *mosA* transcript begins upstream from the original annotation (Figure 5). Promoter and ORF predictions (BProm, FGENESB; http://linux1.softberry.com/berry.phtml) for *mosA* were consistent with the 5′ RACE results. We constructed a new arabinose-inducible *mosA* vector (pMosA) that included the entire *mosA* transcript; induction of *mosA* expression from this vector completely blocked the growth inhibitory effect of MosT in *E. coli* or *E. coli* harboring *mosAT* SXT (Figure 4A–C). An empty pBAD18 vector control did not diminish *mosT* toxicity (Figure 4A–C). Together, these findings are consistent with the idea that *mosT* and *mosA* encode a toxin and antitoxin respectively.

### Excision Promotes *mosAT* Transcription

**Figure 6:**
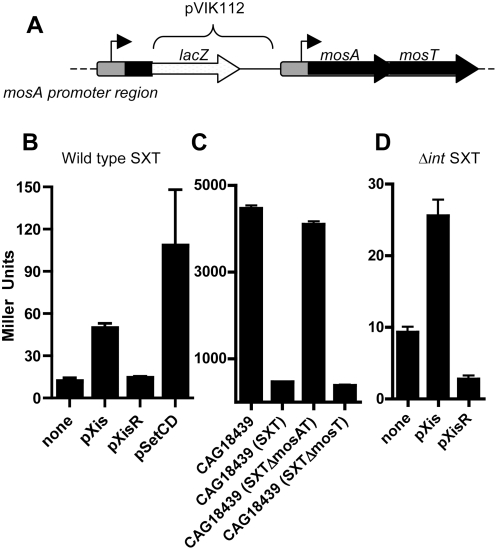
Influence of Xis and SetCD on *mosA* expression. A) Schematic of chromosomal *mosA*:: *lacZ* transcription reporter within the *mosAT* locus. Thick black arrows represent ORFs as predicted by bioinformatics and the thin arrow represents the predicted location of the *mosA* promoter. B, D) β-galactosidase activities of the chromosomal *mosA::lacZ* fusion in CAG18439 containing wt SXT (B) or in CAG18439 containing Δint SXT (D) along with the indicating expression vectors. C) β-galactasidase activities from a plasmid-borne *mosA-lacZ* fusion (in pPmosA) in the indicated strains. All β-galactasidase measurements were conducted on 15 hr cultures and the results shown are the means and standard deviation from at least 9 independent cultures.

To begin to explore the factors that promote expression of the *mosAT* locus, we inserted a promoterless *lacZ* gene downstream of the *mosA* promoter, leaving intact copies of the wild type genes (Figure 6A). *mosA* and *mosT* are likely co-transcribed since the two genes overlap, additional 5′ RACE experiments and Northern analyses are consistent with this idea (data not shown), and other characterized TA loci are operons [33],[34]. There was relatively weak basal activity of the *mosA* promoter measured in an overnight culture of the reporter strain (Figure 6B) and no detectable β-galactosidase activity in an isogenic control strain lacking a *lacZ* fusion (data not shown).

We explored if the low activity of the *mosA* promoter was due to repression by MosA, since such autorepression has been observed for several other TA loci [33],[34]. The β-galactosidase activity derived from a plasmid-borne *mosA* promoter-*lacZ* fusion (in pPmosA) was found to be ∼10-fold higher in the absence vs. the presence of SXT, suggesting that SXT encodes a repressor of *mosA* (Figure 6C). Furthermore, reporter activity was elevated to a similar extent in SXT^−^ and *mosAT* SXT *E. coli* strains, and reduced to comparable levels in strains containing wild type and *mosT* SXT (Figure 6C). Together, these data indicate that MosA can function independently of MosT to repress its own expression.

Since SXT is vulnerable to loss following its excision, we tested if factors that promote SXT excision also promote *mosAT* expression. Overexpression of Xis from the arabinose-inducible promoter in pXis augmented the activity of the chromosomal *mosA-lacZ* fusion more than 4-fold (Figure 6B), whereas addition of arabinose to the reporter strain harboring the control vector, pXis-R did not alter β-galactosidase activity (Figure 6B). Furthermore, overexpression of SetC and SetD, the key transcription activators that control SXT transfer, increased transcription from the *mosA* promoter (Figure 6B). These observations suggest that expression of *mosAT* is linked to conditions, such as production of Xis, SetC and SetD, that promote SXT excision. Furthermore, elevations in SXT copy number that may occur with excision [20] would increase *mosAT* gene dosage, providing more template for their transcription.

To test if SXT excision was essential for Xis-mediated induction of *mosA* transcription, the chromosomal *mosA-lacZ* fusion was introduced into an *int* SXT background. In this context, overexpression of Xis still increased expression of *mosA* (∼2.5 fold) but not to the same extent as in the wt SXT background (∼4.0 fold) (compare Figure 6B and 6D). Since Xis can augment *mosA* expression independently of SXT excision, it is possible that this RDF can directly influence transcription, as described for several other RDFs [35].

## Discussion

When integrated in a host genome, SXT and other ICEs are stably maintained, as they are replicated along with the chromosome. However, once excised from the host genome, even with some degree of replication, ICEs are at risk of being lost if cell division occurs prior to element re-integration. Without mechanisms to promote SXT reintegration and/or prevent growth of daughter cells lacking SXT, cells that have lost SXT should be detectable, since SXT excises from the chromosome in more than 1 in 100 cells. Yet our previous attempts to identify cells that have lost SXT using replica plating-based assays did not reveal any SXT loss. In light of our current work, our previous failure to detect SXT loss is understandable. Using the positive selection-based strategy developed here, we detected SXT loss in only 1 in 10^7^ cells. Furthermore, SXT appears to be lost after the ICE excises from the chromosome, since loss doesn't occur when SXT cannot excise. Two genes that lack similarity to genes of known function were found to promote SXT maintenance, although they do not influence SXT excision frequency or conjugation frequency (data not shown). These 2 genes, *mosT* and *mosA*, function as a toxin-antitoxin system. Exogenous expression of *mosT* greatly impaired cell growth, and *mosA* expression ameliorated MosT toxicity. Basal expression of *mosAT* is quite low; however, factors that promote SXT excision, like Xis and SetCD, augment *mosAT* expression. Thus, our observations suggest that when the element is extrachromosomal and vulnerable to loss, SXT activates a TA module to minimize the formation of SXT-free cells.

Comprehensive screens for genes that promote ICE maintenance have not previously been carried out and explorations of mechanisms that promote ICE stability have received little attention. To date, there has been only one report of a gene that promotes the stability of an ICE. Qui et al. found that the *P. aeruginosa* ICE PAPI-1 contains a homologue of the plasmid and chromosome partitioning gene *soj* (*parA*). They demonstrated that deletion of the *soj* homologue from PAPI-1 resulted in complete loss of PAPI-1 from *P. aerguinosa* [3]. The mechanism by which Soj promotes PAPI-1 maintenance remains to be elucidated; however, it is unlikely that Soj functions as a toxin like MosT.

Prior to our study, TA systems have not directly been shown to promote ICE maintenance. Dziewitt et al. proposed that *s044* and *s045*, two SXT genes with similarity to TA genes found in the *Paracoccus aminophilus* plasmid pAMI2, promote SXT stability [23]. They found that these two SXT genes augmented the stability of a heterologous unstable plasmid [23]; however, our observation that deletion of *s044* and *s045*, (in the Δ7 SXT mutant), did not result in elevated SXT loss argues against the idea that these genes influence SXT maintenance.

Unlike previously described TA loci, *mosAT* are part of a dynamic mobile genetic element that is predominantly found integrated in the chromosome but that can also exist in an extrachromosomal form. As demonstrated by the *lacZ* transcriptional fusion data (Figure 6), *mosAT* has low basal transcription due to autorepression by MosA, as described in several other TA loci [33],[34]. Therefore, *mosAT* likely remains in a repressed state while SXT is integrated in the host chromosome. It is unknown whether these genes have physiologic function when SXT is integrated. However, *mosAT* appear to be derepressed by the actions of Xis and SetC and SetD, proteins that promote SXT excision. Thus, the activity of *mosAT* may be limited to the time when SXT is extrachromosomal and vulnerable to loss. Since our current studies are population-based, a more refined understanding of the timing of *mosAT* expression in relation to SXT excision will be possible if we can develop assays to monitor *mosAT* expression and SXT excision in single cells.

There is controversy about the ordinary physiologic role of toxins that are part of chromosomal toxin antitoxin systems. Some propose that these toxins' physiologic function is to kill host cells whereas others propose that toxins function to limit metabolic processes such as protein synthesis as part of stress responses [29],[36]. MosT could act in either fashion to promote the maintenance of the excised form of SXT. Assuming that *mosAT* is an operon and MosA is less stable than MosT, then cells from which SXT is absent should be subject to the effects (presumably death) of MosT, similar to many plasmid-encoded toxins that are thought to kill host cells that fail to inherit the plasmid. However, it also possible that MosT is active prior to loss of SXT (e.g., via preferential degradation of MosA, as described for the RelB anti-toxin, [29]), and that it stalls cell growth until SXT has reintegrated and MosT's effects can be turned off. If so, MosT's effects would need to be reversible, so that the cell could resume growth upon SXT re-integration. Future studies to discover the MosT target and its mechanism(s) of action may enable discrimination between these possibilities.

If *mosAT* guards against SXT loss when the element is extrachromosomal, as we propose, then linking expression of these genes to SXT excision makes sense. There are several reports of excised forms of mobile elements increasing expression of genes that act on the excised (and often circular) forms of the elements. For example, expression of the PAPI-1 *soj* gene is increased in the circular form of the ICE by the joining of a promoter located on the 3′ end of the integrated element to *soj* (which is found at the 5′ end of the ICE) when PAPI-1 circularizes [3]; a similar mechanism drives the expression of genes required for conjugative transfer of Tn*916* [37]. The *mosAT* locus is found near the middle of the SXT genome, so generation of a promoter through SXT circularization cannot directly influence *mosAT* expression. Instead, we found that factors that promote SXT excision including Xis and SetC and SetD augmented *mosAT* expression. Xis may act, at least in part, directly at the *mosA* promoter to increase *mosAT* expression, since Xis augmented *mosA* expression even when SXT was unable to excise. Other RDFs such as the Cox proteins HP1 and P2, have also been shown to act as transcription factors [38]. Currently the factors that govern Xis expression and activity are not known. It is also unclear at this point whether SetC and SetD act directly to promote *mosAT* expression.

Although *mosAT* expression and function appears to be highly adapted to SXT, these genes are not found in all SXT/R391 family ICEs. A few of these elements, including R391, an ICE originally derived from an African *Providencia rettgeri* strain [8],[15], and ICE*Vch*Mex1, an ICE derived from a Mexican environmental *V. cholerae* strain [39], lack *mosAT*. The *mosAT* locus is found in one of the SXT ‘hot-spots’, regions of hypervariability that are not conserved among SXT/R391 family elements (Beaber et al 2002). As such, *mosAT* does not represent a universal strategy for the maintenance of the stability of SXT/R391 family ICEs. Preliminary studies suggest that R391 is only slightly less stable than SXT, raising the possibility that R391, like SXT, has acquired additional genes to promote its maintenance.

A recent BLASTP search revealed many proteins with sequences similar to MosA and MosT in gram-negative bacteria. Overall, MosT exhibited greater conservation and contains a widely conserved domain of unknown function (DUF1814) present in both bacteria and archaea. However, MosA homologues were routinely found adjacent to MosT homologues. In total, there were 5 proteins whose amino acid sequences are identical or nearly identical to MosT from SXT and 30 proteins whose amino acid sequences bear significant similarity to MosT (E values less than 4×10^−50^). Proteins with near identity to MosT, from several recently sequenced *V. cholerae* strains and from *Proteus mirabilis* HI4320, appear to be part of previously unannotated SXT/R391 family elements. However, with one exception, the proteins with significant similarity to MosT appear to be encoded by chromosomes that do not contain an SXT/R391 family ICE, raising the possibility that they function to promote the stability of a non-SXT-related ICE or perhaps perform another function for the host genome. There is an octopine type Ti plasmid from *Agrobacterium tumefaciens* that contains genes with similarity to *mosA* and *mosT*, suggesting that these genes may be able to promote the stability of plasmids as well as ICEs. Overall, the presence of *mosAT*-like genes outside of SXT/R391 family of ICEs suggests that these genes have been and perhaps remain transmissible.

## Materials and Methods

### Media, Growth Conditions, and Growth Assays

Bacterial cultures were routinely grown in Luria-Burtani (LB) broth at 37°C in a shaking incubator. Antibiotics were used at the following concentrations: ampicillin, 100 µg/ml; chloramphenicol, 20 µg/ml; kanamycin, 50 µg/ml; nalidixic acid, 40 µg/ml; sulfamethoxazole, 160 µg/ml; trimethoprim, 32 µg/ml; tetracycline, 6 µg/ml; and spectinomycin 30 µg/ml. 5-bromo-4-chloro-3indolyl-beta-D-galactopyranoside (X-gal) was used at 60 µg/ml, arabinose at 0.02% (vol/vol) and glucose at 0.2% (vol/vol). Strains were maintained at −80°C in LB containing 20% (vol/vol) glycerol.

For the growth curves presented in Figure 4, cultures were initially grown overnight in the presence of appropriate antibiotics plus 0.2% glucose. The cells were then washed 2× in equal volumes of fresh LB and then diluted 1∶1000 into LB plus 0.02% arabinose or 0.2% glucose. Each culture was tested in triplicate by applying 200 µl to each of 3 wells of a 96-well culture plate. The cultures were then grown in a Synergy HT microplate reader (BioTek) with shaking at 37°C for10 hours; OD600 measurements were acquired every 20 min. The numbers of colony-forming units were measured by plating serial dilutions of cultures grown under similar conditions on plates containing chloramphenicol and 0.2% glucose.

### Strain and Plasmid Construction

A complete list of the strains and plasmids used in this study can be found in Table 4.

**Table 4:** Strains and plasmids used in this study.

Strain or plasmid	Genotype or phenotype	Reference or source
MG1655	*F-λ*, *ilvG*, *rfB50*, *rpn1*	[46]
CAG18439	MG1655 *lacZU118 lacI42:Tn10*	[47]
BW25113	*lacIq*, Δ*lacZ*, Δ*araBAD*, Δ*rhaBAD, hsdr, rrnB*	[24]
HK57	F-, Δ*lacU169*, *araD 139*, *rbsR*, *rpsL*, *thiA*, *relA*, *secB::*Tn*5*, *malT^c^malE*18-1, *srl::*Tn*10, recA1*	[41]
HW220	SXT+ exconjugant of CAG18439	[19]
RF146	HW220 Δ*lacI::kn*, Δ*galETKM::P_lac_aad7*, 3′*traG*ΩpFRTIq	This study
RF160	RF146 Δ*int*	This study
RF184	RF146 Δ*rum′B-rumA* (Δ1)	This study
RF210	BW25113 Δ*lacI::kn*, Δ*galETKM::P_lac_aad7*, 3′*traG*ΩpFRTIq Δ*s024-s040* (Δ2)	This study
RF186	RF146 Δ*traI* (Δ3)	This study
RF212	RF146 Δ*traD-s043* (Δ4)	This study
RF280	RF146 Δ*s044-s045* (Δ7)	This study
RF224	RF146 Δ*traL-traA* (Δ8)	This study
RF147	RF146 Δ*s052-traN* (Δ9)	This study
RF163	RF146 Δ*s060-s073* (Δ10)	This study
RF583	RF146 Δ*s074-traG* (ΔD)	This study
RF584	RF146 Δ*eex-s086* (ΔE)	This study
RF291	RF146 Δ*s052-s053* (ΔB)	This study
RF293	RF146 Δ*traC-traN* (ΔA)	This study
RF335	RF146 Δ*mosT*	This study
RF336	RF146 Δ*s054*	This study
RF413	RF146 *recA1*	This study
RF414	RF335 *recA1*	This study
RF560	RF335 Δ*int*	This study
RF366	RF146 that subsequently lost SXT*3′traG*ΩpFRTIq	This study
RF377	SXT*3′traG*ΩpFRTIq exconjugant of RF366	This study
RF573	SXTΔ*mosT 3′traGΩ*pFRTIq exconjugant of RF366	This study
RF404	RF335 that subsequently lost SXTΔ*mosT 3′traG*ΩpFRTIq	This study
RF428	SXT*3′traG*ΩpFRTIq exconjugant of RF404	This study
RF429	SXTΔ*mosT 3′traG*ΩpFRTIq exconjugant of RF404	This study
RF503	CAG18439 SXTΔ*florR::kn*	This study
RF513	RF503 Δ*mosT-mosA*	This study
RF514	RF503 Δ*mosT*	This study
RF561	HW220 P*_mosA_*ΩpVIK112	This study
RF567	HW220 Δ*int*, P*_mosA_*ΩpVIK112	This study
pBAD33	Cm^R^, arabinose-inducible vector	[32]
pBAD18	Amp^R^, arabinose-inducible vector	[32]
pBAD-TOPO	Amp^R^, arabinose-inducible vector	Invitrogen
pAH162	Tet^R^, suicide vector	[40]
pKD4	Kn^R^ PCR template for one-step chromosomal gene activation	[24]
pVIK112	Suicide vector containing lacZ	[42]
pCB192	LacZ expression vector	[43]
pMosT	pBAD33 containing *mosT*	This study
pMosT′	pMosT containing *mosT* ORF with a stop codon at amino acid 11	This study
pMosA	pBAD18 containing *mosA* ORF through +1 of transcription	This study
pPmosA	pCB192 containing promoter region of *mosA*	This study
pSetCD	pBAD-TOPO containing *setCD*	[18]
pXis	pBAD-TOPO containing *xis*	[20]
pXis-R	pBAD-TOPO containing *xis* in the reverse orientation	This study

In the SXT loss reporter strains, Δ*lacI::kn* and Δ*galETKM::P_lac_spec* were introduced into the respective chromosomal loci using the lambda Red recombination system as described [24]. The PCR product used to create the Δ*lacI::kn* deletion/insertion was generated using the pKD4 plasmid as a template and primers designed to omit the FRT sites. To generate the P_lac_spec PCR product used to create the Δ*galETKM::P_lac_spec* deletion/insertion, first a spectinomycin cassette was cloned into pCRII-TOPO (Invitrogen); then, this fragment along with the upstream *lac* promoter was used as a template to amplify the P_lac_spec fragment. The structure of the engineered Δ*lacI::kn* and Δ*galETKM::P_lac_spec* loci were each confirmed by several PCR assays. The construct used to insert *lacI^q^* into SXT (pFRTIq) was constructed by first cloning *lacI^q^* and the FRT sequence (GAAGTTCCTATACTTTCTAGAGAATAGGAACTTC) into the suicide vector pAH162 [40]. A FRT site was also introduced into *E. coli* CAG18439 carrying SXT immediately downstream of *traG* using lambda Red-based technology in all strains except ΔD and ΔE. Finally, pFRTIq was introduced into this strain and the Flippase encoded on plasmid pCP20 [24] was used to mediate the integration of pFRTIq into SXT immediately 3′ of *traG*. In ΔD and ΔE, a FRT site was initially introduced into the site of the deletion and then pECA102 (kindly provided by D.E. Cameron), which contains a modified Flippase, was used to mediate the integration of pFRTIq. The correct insertion of pFRTIq was verified using PCR.

RecA mutants were created by P1 transduction of the *recA* allele from HK57, an *E. coli* strain that contains the RecA1 allele linked to *slr*::Tn10 [41]. Transductants were selected as tetracycline-resistance cfu and the *recA* mutation was subsequently confirmed by assessing sensitivity to UV exposure.

The arabinose-inducible *mosT* and *mosA* expression vectors, pMosT and pMosA, were constructed by amplifying *mosT* and *mosA* with flanking restriction sites using PCR and then cloning the PCR products into pBAD33 and pBAD18 [32], respectively. The correct inserts were verified by PCR as well as DNA sequencing.

A chromosomal *mosA::lacZ* fusion was constructed by initially amplifying a fragment encompassing the predicted *mosA* promoter. Then, the PCR product was cloned into the suicide vector, pVIK112, which contains a *lacZ* gene downstream from a multiple cloning site [42]. Integration of the plasmid into the chromosome enabled measurement of *mosA* promoter activity in the presence of an intact copy of the gene.

pPmosA was created by subcloning the mosA promoter region into the *lacZ* fusion vector, pCB192 [43] in order to evaluate the expression of the *mosA* promoter in the absence of SXT.

### SXT Loss Calculation

Reporter strains were generally grown for 15 hr in LB and then serial dilutions were plated on agar containing spectinomycin and kanamycin. Approximately 100–500 colonies were subsequently replica plated or patched to agar containing chloramphenicol, a marker carried by SXT, to estimate the false positive rate. The false positive rate was calculated by dividing the number of CmR cfu by the number of SpecR cfu. Thus, the frequency of SXT loss was calculated as (Spec^R^ cfu/Total cfu)*(1-frequency of false positives). All loss assays were repeated a minimum of 6 times. A two-tailed, non-paired T-test was used to assess the statistical difference between SXT loss values. P values≤0.05 were considered statistically different.

### Conjugation Assay

Conjugation experiments were conducted as previously described [13]. Briefly, donor and recipient cells were mixed on a LB plate for 2 hours at 37°C. The cells were then resuspended and plated on the appropriate selective media to count the number of donor, recipient and exconjugant cfu. In order to accurately measure loss in the context of mating only, strains were kept on selection for SXT until the start of the experiment. Prior to mixing donor and recipient strains, the cells were washed 2× in fresh LB to remove traces of antibiotics from the liquid media.

### Miscellaneous Molecular Biology Methods

DNA manipulations were carried out using standard techniques [44]. Quantitative Real Time PCR assays were conducted as previously described [20] using primers to amplify *attB*, and a portion of the *3′* region of *prfC*. DNA sequencing was done by GeneWiz. 5′RACE was conducted as per the manufacturer's instructions (Invitrogen) to determine the start site for *mosA* transcription. Assays for β-galactosidase activities in overnight cultures were carried out as previously described [45].
